# Protocol for the enhanced analysis of electrophysiological data from high-density multi-electrode arrays with *nicespike* and *spikeNburst*

**DOI:** 10.1016/j.xpro.2025.104195

**Published:** 2025-11-21

**Authors:** Robert Wolff, Alessia Polito, Alessio Paolo Buccino, Michela Chiappalone, Valter Tucci

**Affiliations:** 1 Genetics and Epigenetics of Behavior (GEB) Laboratory, Istituto Italiano di Tecnologia (IIT), 16163 Genoa, Italy; 2 Padova Neuroscience Center (PNC), University of Padova, 35129 Padua, Italy; 3 Allen Institute for Neural Dynamics, Seattle, WA 98109, USA; 4 Department of Informatics, Bioengineering, Robotics, and Systems Engineering (DIBRIS), University of Genova, 16145 Genoa, Italy

**Keywords:** Bioinformatics, Cell Biology, Neuroscience, Biotechnology and bioengineering

## Abstract

High-density multi-electrode arrays enable the recording of *in vitro* neuronal activity with exceptional spatial and temporal resolution. Here, we describe a protocol for analyzing these extensive datasets by using two complementary tools. The *nicespike* tool implements a full electrophysiological data analysis pipeline featuring graphics processing unit–accelerated spike sorting via template matching with *Kilosort*, enabling accurate identification of neuronal units across multiple electrodes. The *spikeNburst* tool incorporates advanced techniques for spike train filtering, burst and network burst detection, and synchronization analysis.

## Highlights

• Instructions for setting up graphical user interfaces for HD-MEA data analysis
• Instructions for analyzing voltage traces from HD-MEA recordings using spike sorting
• Procedures for characterizing spike-sorted units and neural network dynamics
• Guidance on extending the analysis for HD-MEA devices from various manufacturers

## Abstract (editor highlights)

Publisher’s note: Undertaking any experimental protocol requires adherence to local institutional guidelines for laboratory safety and ethics.

## Graphical abstract

**Figure:**
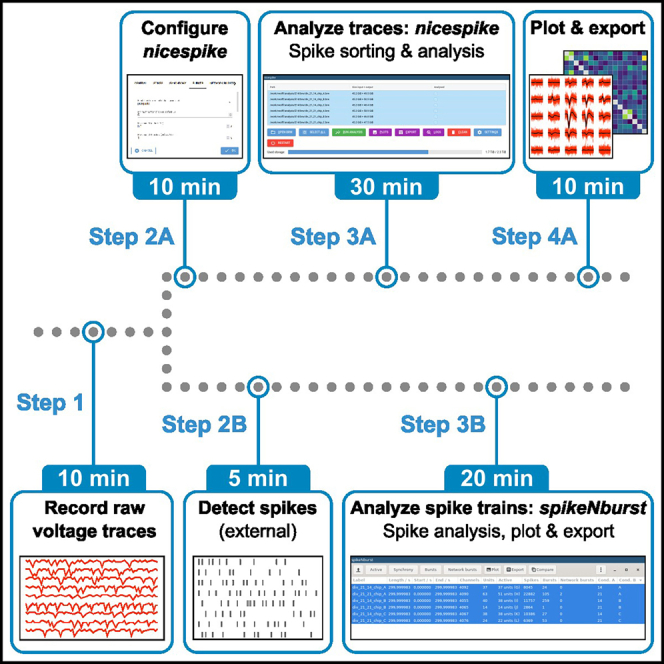

## Before you begin

This protocol outlines the steps for analyzing raw voltage recordings of extracellular electrophysiological activity from high-density multi-electrode array (HD-MEA) systems with exceptional spatial and temporal resolution,^1^^,^^2^^,^^3^ integrating state-of-the-art spike sorting, burst detection, and synchrony measures within an accessible and flexible framework. It was adapted to analyze *in vitro* neuronal culture recordings from the 3Brain device, which comprises 4,096 electrodes. However, we highlight the versatility of the protocol for analysis of recordings from different HD-MEAs by minimal adaptations of the open-source software.

The spiking activity of individual neurons is detected by a process called *spike sorting*,^4^^,^^5^^,^^6^^,^^7^^,^^8^ which, applied on HD-MEA data, has resulted in advances in the characterization of neuronal growth, connectivity, and plasticity.^2^^,^^9^^,^^10^ The demand of fast, reliable, and reproducible HD-MEA results is still unmet. Analyzing these extensive datasets presents challenges, such as artifact removal, spike sorting, and accurate assessment of neuronal synchronization.^11^^,^^12^ Moreover, the management of spike sorting methods is time-consuming and technically complex, resulting in limited user access and loss of data reproducibility. Existing software solutions, including the default tools integrated within standard analytical platforms, such as Brainwave^13^ from 3Brain, primarily provide fundamental spike detection functionalities which are restricted to single-channel analyses. This limitation often results in overestimation of neuronal units and network activity due to redundant measurements of neurons being simultaneously recorded by multiple electrodes. Furthermore, while effective for systems with few electrodes, analysis tools such as Spycode^14^ and MEA-NAP^15^ are neither scalable nor compatible with contemporary HD-MEA datasets.

### Innovation

To address these existing challenges, we introduce a structured protocol for conducting comprehensive and user-friendly HD-MEA analyses with two python-based tools: ‘*spikeNburst*’ and ‘*nicespike*’. Here, we describe the implementation, features, and application of these tools, demonstrating their efficacy in overcoming the limitations of existing methods while providing a scalable solution for large-scale electrophysiological datasets^16^ ensuring efficient data handling and robust analytical outcomes.

The spikeNburst tool incorporates advanced methodologies for spike train filtering, burst and network burst detection, and synchronization analysis. Complementing this, we have implemented a full analysis pipeline in the nicespike tool, featuring spike sorting via template matching with Kilosort, enabling accurate identification of neuronal units across multiple electrodes. This protocol ensures more precise analyses by reducing redundancy and overestimation inherent in single-channel approaches and enhancing the study of neural network dynamics and single-cell activity in detail. Moreover, both tools offer graphical user interfaces (GUI) and programmatic access as python modules, ensuring accessibility for diverse user needs.

The implemented methods for analyzing HD-MEA recordings, are summarized in Table 1.

**Table 1:** Methods implemented in nicespike and spikeNburst

Description	Tool	References
Reading of electrophysiological recordings using spikeinterface and python-neo	nicespike^a^	Buccino et al.^7^; Garcia et al.^17^
Spike sorting with template matching using Kilosort	nicespike	Pachitariu et al.^18^
Unit filtering using inter-spike-interval (ISI) violation method	spikeNburst^a^^,^^b^	Hill et al.^19^
Burst and network burst detection	spikeNburst^a^^,^^b^	Chiappalone et al.^20^; Pasquale et al.^21^
Network burst leader analysis	spikeNburst^a^^,^^b^	Eytan and Marom^22^; Eckmann et al.^23^
Synchrony: spike distance using burst_sync	spikeNburst^b^	Eisenman et al.^12^; Kreuz et al.^24^
Synchrony: spike-time tiling coefficient (STTC) using burst_sync	spikeNburst^b^	Cutts and Eglen^11^; Eisenman et al.^12^
Synchrony: phase synchrony using burst_sync	spikeNburst^b^	Eisenman et al.^12^; Patel et al.^25^; Patel et al.^26^; Li et al.^27^
Unit spike template characterization	spikeNburst^a^^,^^b^	Bakkum et al.^28^; Connors and Gutnick^29^; Becchetti et al.^30^; Puia et al.^31^

a These methods we re-implemented.

b Analyses performed with spikeNburst are also part of nicespike.

### Institutional permissions

The experiments involving mouse embryonic stem cells (mESCs) were conducted in accordance with institutional guidelines for laboratory safety and ethics of the Fondazione Istituto Italiano di Tecnologia, Italy. The primary mouse embryonic fibroblast (MEF) feeder cells used in this study were derived from C57BL/6J WT mice at the same institution. All animal procedures were approved by the Animal Research Committee and the Veterinary Office of Fondazione Istituto Italiano di Tecnologia, Italy (Authorization code: 175/2022-PR).

### Prepare the neuronal cell cultures: *In vitro* corticogenesis

**Timing: 7 weeks**

The results (see expected outcomes) presented in this protocol are obtained by recording spontaneous electrophysiological activity of wild-type-mESCs-derived cortical neurons.

1. Perform *in vitro*, 2D corticogenesis by adapting a previously described protocol.^32^^,^^33^
  a. Culture E14 mouse embryonic stem cell (mESC) line on laminin-coated dishes and WT-B1 mESCs on mytomicin-treated MEFs feeders in ES medium.
    **CRITICAL:** Control regularly the samples to exclude the presence of mycoplasma, e.g., using the MycoSPY kit.
    ***Note:*** To store the cells, supplement the ES medium with 10% DMSO, then freeze at −80°C overnight, and transfer to − 196°C for long-term storage.
  b. The day before the differentiation, plate the mESCs lines at low density on laminin-coated dishes.
  c. The day after, culture the cells in DDM medium supplemented with B27-without-vitamin-A as previously reported.^33^
  d. From day 0 to 8 of culture, supplement the DDM medium with the dorsomorphin analog DMH1-HCl at 1 μM instead of cyclopamine that has been used previously.^33^
  e. At 12 days of 2D *in vitro* corticogenesis, plate E14 or WT-B1 neuroprogenitor cells on 0.1 mg/ml PEI and 33 μg/ml laminin coated Accura chips (3Brain) according to the manufacturer’s instructions.
  f. Differentiate until 21 days *in vitro* (DIV 21), and maintain in complete Neurobasal medium for another 21 days.
    ***Note:*** See materials and equipment for complete media recipes with sources in the key resources table.
    **CRITICAL:** During differentiation, keep the cell cultures at 37°C under an atmosphere of 5% CO_2_. Filter the complete medium with a bottle-top filter (0.22 mm) and store at 4°C. Use within 1 week.

### Record raw voltage traces and detect spikes

**Timing: 10 min per recording**

The voltage traces used in the results presented in this protocol are recorded using Accura recording chips with 4,096 micro-electrodes and an active area of 3.8 mm × 3.8 mm. We recorded spontaneous activity during 5 min.

***Note:*** You may record activity for longer duration, but storage space and computing memory (in subsequent analyses) might be an issue for longer recordings.

2. Record spontaneous electrophysiological activity at two weeks (DIV 21+14) and at three weeks (DIV 21+21) after the end of the differentiation.
  a. Acclimatize the culture in the BioCAM DupleX system for 2 min.
  b. Record for 5 min the spontaneous activity using Brainwave software.
    **CRITICAL:** We performed the recordings in raw mode, which saves the full raw voltage traces. Despite potential information loss in sparse event based recordings, the tools of this protocol experimentally support their analysis. For subsequent spike sorting it is necessary to choose a high sampling rate, e.g., 20 kHz.
    ***Note:*** In this protocol, we recorded electrophysiological activity from 2D cultures by applying the preset “Neuronal culture” as model. The hardware high-pass filter was set at 10 Hz. Adapt these settings according to manufacturer’s instructions matching your samples.
  c. For the comparison analysis presented in this protocol, detect the spikes and bursts from the raw voltage traces (brw files), which are saved to bxr files, using the Brainwave software.

### Prepare the nicespike tool

**Timing: 10 min**

The computer used with the nicespike tool for the results presented in this protocol is equipped with an Xeon W-2235 CPU, 256 GB DDR4 (3200MHz) RAM and a CUDA-accessible Quadro RTX5000 graphics processing unit (GPU), and is running on Ubuntu 20.04. The following steps describe the installation of nicespike using docker. It uses a docker container, provided by spikeinterface, that includes Kilosort 2. An alternative way of installation in a python-venv environment is described at https://codeberg.org/spiky/nicespike.

**CRITICAL:** Make sure you have git, docker and CUDA properly installed (see key resources table).

3. Install nicespike.
  a. Open a Linux terminal for running the commands in the next step.
  b. Download the code for docker container creation and change into the directory:
    git clone -b docker --single-branch https://codeberg.org/spiky/nicespike.git
    cd nicespike
  c. Select or create an output directory where the analysis results will be stored:
    mkdir output
  d. Edit the user and group identifier inside the file docker-compose.yml to match the used Linux system set-up by modifying the fields UID (line 5) and GID (line 6) to the ones of the running user.
    ***Note:*** You can obtain these identifiers by executing the command id inside the terminal.
    ***Note:*** You may add an extra Linux user for the purpose of running nicespike.
  e. Edit the volumes inside the file docker-compose.yml, line 12 to include the output directory created in the previous step (first path in line 12) and data input directories (add lines after line 12).
    **CRITICAL:** Make sure that the user with the identifiers specified in the previous step has read access to the input directories and read-write access to the output directories specified.
    ***Note:*** See the docker documentation on volumes for detailed instructions.
  f. Optionally, edit the host port inside the file docker-compose.yml, line 10 to your preferred (first number in line 10).
    ***Note:*** See the docker documentation on networking for detailed instructions.
  g. Build, compose and run the docker image with:
    docker compose up --detach
    ***Note:*** This will start the nicespike tool, accessible in a web browser at http://localhost:8080 if the port was not changed in step f, else replace 8080 by the specified port.

### Prepare the spikeNburst tool

**Timing: 5 min**

The following steps are necessary only if the spikeNburst tool is used with externally detected spikes. An alternative way of installation in a python-venv environment is described at https://codeberg.org/spiky/spikenburst.

4. Install spikeNburst.
  a. Open a Linux terminal for running the commands in the next steps.
  b. The recommended way is to create and activate a conda environment:
    conda create -n spikenburst cython h5py librsvg matplotlib numpy openpyxl pandas pip python scipy conda-forge::gtk3 conda-forge::odfpy conda-forge::pygobject
    conda activate spikenburst
    **CRITICAL:** It might be necessary to install additional packages for the GUI (GTK 3.0).
  c. Install spikeNburst using pip:
    pip install --extra-index-url https://codeberg.org/api/packages/spiky/pypi/simple/ spikenburst[full]

### Adapt nicespike and spikeNburst for inputs from different recording systems

**Timing: 1 day, depending on the user’s experience**

The following steps explain briefly how to adapt nicespike and spikeNburst for analyzing inputs from different recording systems.

***Note:*** These steps require significant knowledge in python programming and setting up a development environment. We recommend an editable installation with pip from the checked out git repositories for development.

5. Introduce new raw voltage recording file formats to the nicespike tool:
  a. Change the way how input files are read in the function SpikeSorting.read_and_filter in src/nicespike/spike_sorting.py, lines 109–123.
    ***Note:*** Many file formats are already supported by the spikeinterface that is internally used here.^7^
  b. The probe layout might need to be adapted in lines 81–85 and the plotting functions in src/nicespike/plotting_helpers.py may need some adaptations, too.
  c. Further, the filtering for files with the brw extension in src/nicespike/__main__.py, lines 50–51 needs to include the extensions of the new file format.
  d. The spikeNburst module might also need some adaptations if the probe layout does not correspond to 64 times 64 electrodes, namely in the 2D plotting function plot_2d in src/spikenburst/plot.py, lines 206–258.
    ***Note:*** Some parameters are hard-coded and may be adapted by changing src/nicespike/spike_sorting.py, lines 36–37.
    ***Note:*** Additionally, because spikeinterface supports many spike sorting algorithms, the sorting may be modified in src/nicespike/spike_sorting.py, lines 188–227.
    ***Note:*** An example adaptation of nicespike for the reading of HD-MEA recordings from Maxwell Biosystems can be found in the branch read_maxwell of the nicespike source repository. Only few lines of code needed to be changed to make a version of nicespike that can be used with these recordings.
6. Add new spike train file formats to the spikeNburst tool:
  a. Some adaptations must be done to the SpikeAnalysis.__init__ function by adding the reading of new formats in src/spikenburst/spike_analysis.py, line 212.
  b. Further, for the spikeNburst GUI, new file extensions must be added to the filter in src/spikenburst/gui/window.py, line 628.

## Key resources table

**Table:** 

REAGENT or RESOURCE	SOURCE	IDENTIFIER
**Chemicals, peptides, and recombinant proteins**		

Laminin	Sigma-Aldrich	L2020
Poly(ethyleneimine) solution (PEI)	Supelco	P3143
Dulbecco’s modified Eagle’s medium (DMEM) W/O NA PYR	Gibco	41965039
Embryonic stem cell FBS, qualified, US origin	Gibco	16141079
Nonessential amino acids (100×)	Invitrogen	11140035
Sodium pyruvate 100 mM	Invitrogen	11360039
2-Mercaptoethanol 14.3 M	Sigma-Aldrich	M3148
Penicillin/streptomycin (5,000 U/ml)	Invitrogen	15070063
Murine leukemia inhibitory factor (LIF) 10 7 U ml-1	ESGRO-Millipore	ESG1107
DMEM/F12, GlutaMAX supplement	Gibco	31331028
B-27 supplement (50×) without vitamin A	Invitrogen	12587010
N-2 Supplement (100×)	Invitrogen	17502048
Bovine Albumin Fraction V (7.5% solution)	Invitrogen	15260037
Dorsomorphin homolog DMH1-HCl (10 mg)	Tocris	4126
Neurobasal medium	Gibco	21103049
L-glutamine 100×	Invitrogen	25030081
Dimetil solfossido (DMSO)	Sigma-Aldrich	D2438

**Critical commercial assays**		

MycoSPY Master Mix	Duotech/Biontex	M020-050

**Deposited data**		

Example datasets	This paper, G-Node	https://gin.g-node.org/spiky/Wolff_et_al_2025_spiky_data; https://doi.org/10.12751/g-node.9sypk7

**Experimental models: Cell lines**		

Mouse embryonic stem cell line ES-E14TG2a	Present in our laboratory	RRID:CVCL_9108
Mouse embryonic stem cell line WT-B1	The use was gently authorized by Prof. K. John McLaughlin, Columbus US, and the cells were provided by Dr. T. Bouschet, CNRS, France	N/A
E13.5 Primary mouse embryonic fibroblast cells (MEF) from C57BL/6J mice	Derived in our laboratory	N/A

**Software and algorithms**		

Brainwave (4 and 5) for electrophysiological recording	3Brain	https://3brain.com
Ubuntu (20.04) for setup	Canonical Ltd.	https://ubuntu.com
CUDA toolkit (12.6) for calculating on GPU	Nvidia Corp.	https://developer.nvidia.com/cuda-toolkit
Docker (28.1.1) for setup	Docker Inc.	https://docker.com
Git (2.25.1) for setup	Software Freedom Conservancy, Inc.	https://git-scm.com
Python (3.9.16) as dependency	Python Software Foundation	https://python.org
Spikeinterface (0.102.3) for reading electrophysiological recordings in nicespike	Buccino et al.^7^	https://spikeinterface.readthedocs.io
Nicegui (1.4.23) for nicespike GUI	Zauberzeug GmbH	https://nicegui.io
Numpy (2.0.2) as dependency	Harris et al.^34^	https://numpy.org
Neo (0.14.2) for nicespike	Garcia et al.^17^	https://neuralensemble.org/neo
h5py (3.12.1) for reading data	Colette^35^	https://h5py.org
Odfpy (1.4.1) for exporting data	N/A	https://github.com/eea/odfpy
Openpyxl (3.1.5) for exporting data	N/A	https://openpyxl.readthedocs.io
Matplotlib (3.9.2) for plotting	Hunter^36^	https://matplotlib.org
Pandas (2.3.2) as dependency	The Pandas development team^37^	https://pandas.pydata.org
Scipy (1.13.1) as dependency	Virtanen et al.^38^	https://scipy.org
GTK (3.0) for spikeNburst GUI	GTK Team, GNOME	https://gtk.org
Pygobject (3.48.2) for spikeNburst GUI	N/A	https://pygobject.gnome.org
Burst_sync (1.1.5) for spike synchrony analysis	Eisenman et al.^12^	https://github.com/lneisenman/burst_sync,https://codeberg.org/spiky/burst-sync
Nicespike (1.0.0)	This paper, Codeberg, G-Node	https://codeberg.org/spiky/nicespike; https://doi.org/10.12751/g-node.2jqfxi
SpikenBurst (1.0.0)	This paper, Codeberg, G-Node	https://codeberg.org/spiky/spikenburst; https://doi.org/10.12751/g-node.5n7v18
Cytoscape (3.10.1) for expected outcomes	Cytoscape Consortium	https://cytoscape.org
GraphPad Prism (10.5.0) for expected outcomes	Dotmatics	https://graphpad.com

**Other**		

Accura 2D chip	3Brain	https://3brain.com
BioCAM DupleX	3Brain	https://3brain.com
Computer (Precision 5820 Tower Workstation)	Dell	https://dell.com
CPU (Xeon W-2235)	Intel	https://intel.com
RAM (256 GB 3200 MHz DDR4 ECC)	SK Hynix	https://skhynix.com
CUDA-accessible GPU (Quadro RTX5000) for spike sorting	Nvidia	https://nvidia.com

## Materials and equipment

**Table:** 

Media reagents	Final Concentration
**ES medium**	

DMEM	To a final volume
FBS	15% (vol/vol)
Non-essential amino acids	0.1 mM
Sodium pyruvate	1 mM
2-mercaptoethanol	0.1 mM
Penicillin/streptomycin	50 U/ml
LIF	1000 U/ml

**DDM medium**	

DMEM/F12 + GlutaMAX	To a final volume
N2 supplement	1×
Non-essential amino acids	0.1 mM
Sodium pyruvate	1 mM
BSA	500 μg/ml
2-mercaptoethanol	0.1 mM
Penicillin/streptomycinPenicillin/streptomycin	50 U/ml

**Neurobasal medium**	

Neurobasal	To a final volume
L-glutamine	2 mM
Penicillin/streptomycin	50 U/ml
B27 supplement without vitamin A	1×

## Step-by-step method details

### Configure nicespike

**Timing: 10 min**

The settings of the nicespike tool can be changed by the following steps, or the default settings may be used. Setting changes are persistent.

**Figure 1:**
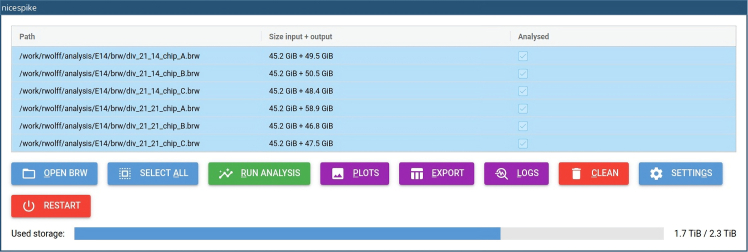
GUI of nicespike after analyzing recording files

1. Access the nicespike tool in a web browser at http://localhost:8080 or with another port depending on the nicespike set-up (Figure 1) and open the settings dialog box by clicking the button labeled ‘SETTINGS’.

***Note:*** For each setting detailed help is obtained by hovering with the mouse pointer over the setting. See Table 2 for a reference of all parameters that can be set.

**CRITICAL:** Some settings may be hidden by default and scrolling down may be needed to see all.

**Table 2:** Adjustable parameters

Parameter	Default	Description	References
**General**			

Number of parallel processes	1	The number of parallel processes, if 1, then no parallelization is used.	–
Export format	ods	The file format used for the tabular export if ‘ods’ or ‘xlsx’. If ‘pkl’ then the analysis object is dumped to a file using the pickle module.	–
Analysis folder	output	The program’s internal output folder used to store the analysis outputs.	–

**Spikes/Active units**			

Use spike-sorted units^a^	true	Whether to use spike-sorted units instead of chip electrodes/channels.	–
Common active channels or units^a^	false	Whether common active channels/units shall be defined on the selected items. If ‘all’ (‘any’) then channels/units are active which have firing rate ≥ minimum in all (any of) selected items.	–
Highpass filter frequency^b^	300 Hz	The highpass filter frequency (onset) used for bandpass filtering.	Buccino et al.^7^
Lowpass filter frequency^b^	6,000 Hz	The lowpass filter frequency (offset) used for bandpass filtering.	Buccino et al.^7^
Bad channels noise threshold^b^	1.7	The threshold for removing channels with noise higher than the threshold times the median noise over all channels.	–
Bad channels constant periods threshold^b^	10	The minimum number of consecutive frames with same value for channels to be removed.	–
Minimum firing rate	0.05 Hz	The minimum firing rate to define the active units.	–
Use valid channels only^a^	true	Whether to use only the valid channels saved in the bxr file.	3Brain^39^
Channel group^a^	–	The name of a predefined group of channels inside the bxr file which shall be used instead of all channels. If the specified group does not exist or the string is empty then all channels are used.	3Brain^39^
Maximum ISI violation ratio	0	The maximum allowed value for the inter spike interval (ISI) violation ratio. If ‘0’ all units are selected as active.	Hill et al.^19^
ISI violation threshold	1.5 ms	The threshold for the calculation of the ISI violation ratio.	Hill et al.^19^

**Synchrony**			

STTC: time window	0.5 s	The time window used in the calculation of the spike time tiling coefficient (STTC) for each spike ±Δ*t*.	Cutts and Eglen^11^; Eisenman et al.^12^
Spike distance: number of bins	120	The number of bins used in the calculation of the spike distance.	Eisenman et al.^12^; Kreuz et al.^24^

**Bursts**			

Burst detection method	pasquale	The method to use for the burst detection. The ‘chiappalone’ algorithm is described by Chiappalone et al. (2005) and the ‘pasquale’ algorithm by Pasquale et al. (2010). ‘chiappalone_isi_threshold’ is a modified version of ‘chiappalone’ using the ISI thresholds calculated as in ‘pasquale’ algorithm. The ‘3brain’ method^a^ reads previously calculated bursts by the Brainwave software.	3Brain^13^; Chiappalone et al.^20^; Pasquale et al.^21^
Minimum number of spikes	5	The minimum number of spikes to form a burst.	Chiappalone et al.^20^; Pasquale et al.^21^
Maximum ISI	0.1 s	The maximum inter-spike interval (ISI) for spikes in one burst for ‘chiappalone’ and ‘chiappalone_isi_threshold’ algorithms.	Chiappalone et al.^20^
Maximum ISI threshold	1.0 s	The maximum allowed ISI threshold for ‘pasquale’ algorithm. If for some channels the calculated ISI thresholds are greater, then fallback for these channels to the ‘chiappalone’ method.	Pasquale et al.^21^
ISI threshold parameter	0.1 s	The parameter for the algorithm choice for ‘pasquale’ algorithm.	Pasquale et al.^21^
Maximum ISI for intra burst peak	0.1 s	The maximum allowed ISI for the intra-burst peak for ‘pasquale’ and ‘chiappalone_isi_threshold’ algorithms.	Pasquale et al.^21^
Bin size of logarithmic ISI histogram	0.1	The bin size for the logarithmic ISI histogram (with base 10) for ‘pasquale’ and ‘chiappalone_isi_threshold’ algorithms.	Pasquale et al.^21^
Window length for ISI histogram filtering	3	The window length for the filtering of the ISI histogram for ‘pasquale’ and ‘chiappalone_isi_threshold’ algorithms.	Pasquale et al.^21^
Order of polynomial for ISI histogram filtering	1	The order of the polynomial for the filtering of the ISI histogram for ‘pasquale’ and ‘chiappalone_isi_threshold’ algorithms.	Pasquale et al.^21^
Minimal distance for peak finding	2	The minimal distance in samples between neighboring peaks for finding the peaks in the filtered ISI histogram for ‘pasquale’ and ‘chiappalone_isi_threshold’ algorithms.	Pasquale et al.^21^
Minimum void parameter	0.7	The minimum void parameter for ‘pasquale’ and ‘chiappalone_isi_threshold’ algorithms.	Pasquale et al.^21^; Selinger et al.^40^

**Network bursts**			

Network burst detection method	pasquale	The method to use for the burst detection. The ‘chiappalone’ algorithm is described by Chiappalone et al. (2005) and the ‘pasquale’ algorithm by Pasquale et al. (2010).	Chiappalone et al.^20^; Pasquale et al.^21^
Minimum number of units	5	The minimum number of units involved in a network burst	Chiappalone et al.^20^; Pasquale et al.^21^
Maximum IBEI	0.2 s	The maximum inter-burst-event interval (IBEI) used to separate intra-network-burst IBEIs from IBEIs between network bursts.	Chiappalone et al.^20^; Pasquale et al.^21^
Minimum separation	−0.01 s	The minimum separation of the network bursts for merging after network burst detection. If less than zero, network bursts are not merged.	–
Maximum IBEI for intra network burst peak	0.2 s	The maximum allowed IBEI for the intra-network-burst peak for ‘pasquale’ algorithm.	Pasquale et al.^21^
Bin size of logarithmic IBEI histogram	0.1	The bin size for the logarithmic IBEI histogram (with base 10) for ‘pasquale’ algorithm.	Pasquale et al.^21^
Window length for IBEI histogram filtering	3	The window length for the filtering of the IBEI histogram for ‘pasquale’ algorithm.	Pasquale et al.^21^
Order of polynomial for IBEI histogram filtering	1	The order of the polynomial for the filtering of the IBEI histogram for ‘pasquale’ algorithm.	Pasquale et al.^21^
Minimal distance for peak finding	2	The minimal distance in samples between neighboring peaks for finding the peaks in the filtered IBEI histogram for ‘pasquale’ algorithm.	Pasquale et al.^21^
Minimum void parameter	0.5	The minimum void parameter for ‘pasquale’ algorithm.	Pasquale et al.^21^; Selinger et al.^40^

**Plotting**			

Plot format	pdf	The file format used for the plot saving, e.g., 'png', 'pdf', 'svg', …	–
Plot settings file	–	The file with additional settings for plotting.	–
Color map	viridis	The name of a color map known to matplotlib. Possible values are e.g., 'rainbow', 'Reds', 'cividis' and 'viridis'.	–
AFR bin size	5 s	The bin size for the average firing rate in seconds.	–
ABR bin size	10 s	The bin size for the average bursting rate in seconds.	–
ANBR bin size	20 s	The bin size for the average network bursting rate in seconds.	–
Plot probe map and unit templates^b^	true	Whether to plot the probe map and unit templates with spikeinterface.	Buccino et al.^7^
Plot wave forms^b^	true	Whether to plot the single-unit wave forms with spikeinterface.	Buccino et al.^7^
Plot single channels^b^	true	Whether to plot the single channel measures with spikeinterface.	Buccino et al.^7^

a Only in spikeNburst

b Only in nicespike.

**Figure 2:**
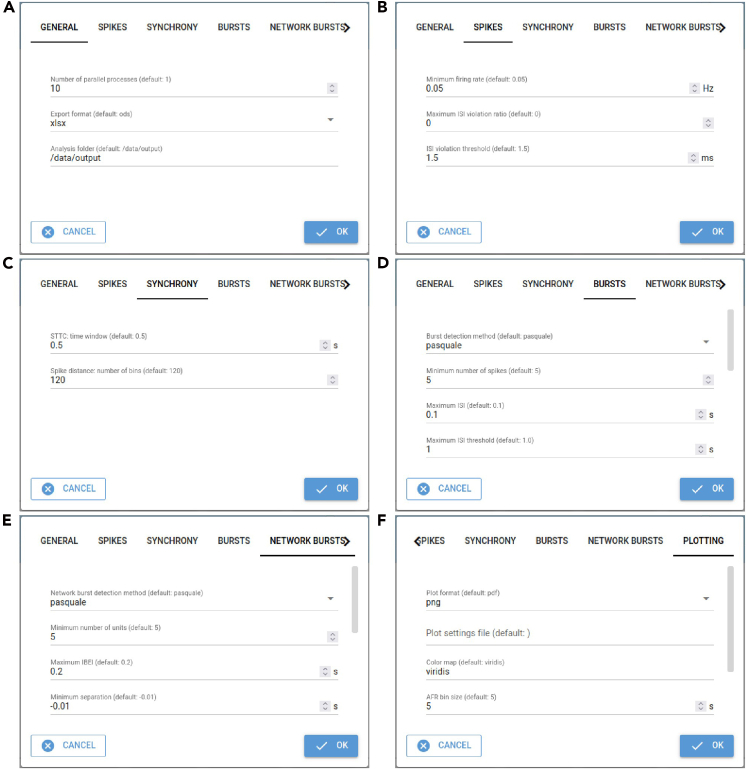
Dialog box tabs for setting parameters in nicespike (A) General, (B) unit filtering, (C) synchrony, (D) burst and (E) network burst detection, and (F) plotting settings.

Troubleshooting: See problem 1.

2. Adjust the general settings (tab ‘GENERAL’, Figure 2A; Table 2, General).

***Note:*** It is recommended to set a higher number of parallel processes to accelerate the analysis. The export format may be changed, if preferred. The analysis folder should not be changed, in particular when nicespike was installed using docker.

3. Adjust the settings for spike detection and filtering (tab ‘SPIKES’, Figure 2B; Table 2, Spikes/Active units).

***Note:*** The minimum firing rate is used both for detection and filtering. If the maximum ISI violation ratio is set to a value greater than 0, the units are filtered using the inter-spike-interval violation method.^19^

**CRITICAL:** Setting the minimum firing rate impacts the template matching. For electrophysiological recordings with very active units, higher minimum firing rates might be appropriate.

4. Adjust the synchrony settings (tab ‘SYNCHRONY’, Figure 2C; Table 2, Synchrony).
5. Adjust the burst detection settings (tab ‘BURSTS’, Figure 2D; Table 2, Bursts).

***Note:*** Depending on the chosen burst detection method, the other parameters below are used or not. The available methods are ‘chiappalone’^20^, ‘pasquale’^21^ (default), and ‘chiappalone_isi_threshold’, which is a modified version of the ‘chiappalone’ method, but using the ISI threshold as calculated within the ‘pasquale’ method.

6. Adjust the network burst detection settings (tab ‘NETWORK BURSTS’, Figure 2E; Table 2, Network bursts).

***Note:*** The available network burst detection methods are ‘chiappalone’^20^ and ‘pasquale’^21^ Optionally, network bursts can be merged by setting the parameter ‘minimum separation’ to a positive value.

7. Adjust the plotting settings (tab ‘PLOTTING’, Figure 2F; Table 2, Plots).
8. Save the settings by clicking on the button labeled ‘OK’.

### Analyze traces with nicespike

**Timing: 30 min per recording**

The following steps explain the automated analysis of raw voltage traces from multiple recordings with nicespike.

9. Load raw voltage recordings:
  a. With the opened nicespike tool, open the file loading dialog box by clicking the button labeled ‘OPEN BRW’.
  b. Navigate to the folder, where the input files are stored and select either the single brw files or folders containing brw files.
  c. Confirm the selection by clicking on the button labeled ‘OK’.
  d. Then, the files are loaded into the application and displayed (Figure 1).

Troubleshooting: See problem 2.

10. Select recordings to be analyzed by pressing Shift key on the keyboard and selecting items with the mouse pointer, or select all by clicking on the button labeled ‘SELECT ALL’.
11. Start the analysis of the selected recordings by clicking on the button labeled ‘RUN ANALYSIS’.

***Note:*** This opens a dialog box which allows reviewing the current status of the analysis of the recording that it is currently processed. Cancellation of the analysis will result in restarting nicespike.

**CRITICAL:** In the case if there is no unit found for a recording, an error message is shown, and the concerned recording is ignored in the further analysis. But, there might be other issues, like insufficient storage space (see also the bottom indicator for the used storage, Figure 1). Thus, it is recommended to study the error messages.

### Plot and export with nicespike

**Timing: 10 min per recording**

After the recordings have been analyzed, automated plots can be created and tables exported by the following steps.

12. Plot the selected analyzed recordings by clicking on the button labeled ‘PLOTS’.

***Note:*** Once the plots are collected and archived, a prompt for saving plots.zip appears. Choose a folder and save the archive.

Troubleshooting: See problem 3, problem 4 and problem 5.

13. Export the selected recordings to tables or python-pickled files (depending on the settings from step 14) by clicking on the button labeled ‘EXPORT’.

***Note:*** Once the files are collected and archived, a prompt for saving export.zip appears. Choose a folder and save the archive.

Troubleshooting: See problem 3 and problem 4.

14. Obtain the log files of the selected recordings for further inspection by clicking on the button labeled ‘LOGS’.

***Note:*** Once the log files are collected and archived, a prompt for saving logs.zip appears. Choose a folder and save the archive.

### Clean analyzed recordings with nicespike

**Timing: 5 min**

The analysis of raw voltage traces from HD-MEA devices can take up a significant amount of storage space. The following steps explain how to clean storage in different ways.

15. To free storage space, analysis outputs can be cleaned from within nicespike by clicking on the button labeled ‘CLEAN’ after selecting recordings (step 19). A dialog box appears with four buttons:
  a. ‘CANCEL’ exits the dialog box without doing anything,
  b. ‘CLEAN’ erases all analysis output but keeps the files in the table,
  c. ‘REMOVE’ drops the selected recordings from the table but keeps the analysis output, and
  d. ‘DISCARD’ erases the big files from the analysis output but keeps the files (plots, etc.) necessary for exporting.

Troubleshooting: See problem 6.

***Note:*** Alternatively, analysis outputs, which are stored in the output directory, can be cleaned manually by using operating system tools.

### Analyze spike trains with spikeNburst

**Timing: 20 min per recording**

The following steps explain how to analyze the spike trains from multiple recordings with spikeNburst.

**Figure 3:**
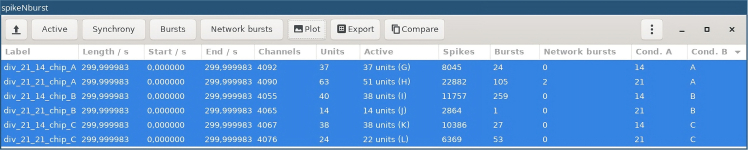
GUI of spikeNburst after analyzing recording files

**Figure 4:**
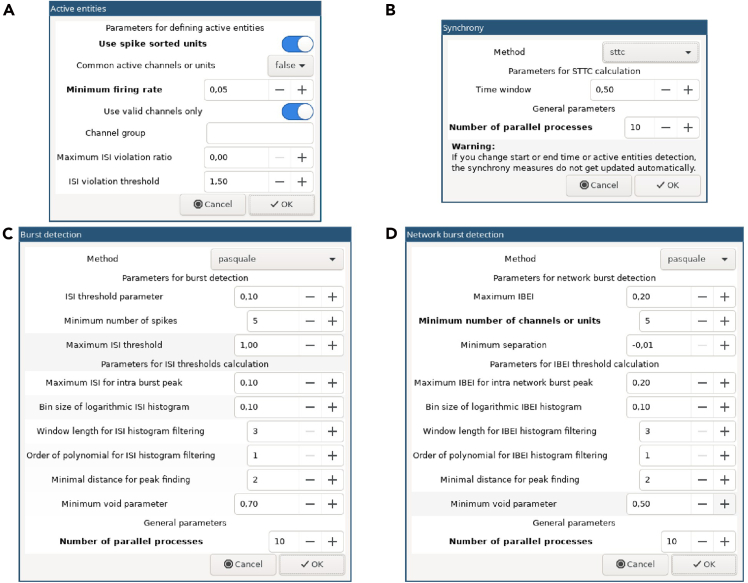
Dialog boxes for setting parameters in spikeNburst (A) Active units, (B) synchrony, (C) burst and (D) network burst detection settings.

16. Open a Linux terminal and run spikeNburst:
  a. Activate the conda environment with.
    conda activate spikenburst
    ***Note:*** This step must be adjusted if you chose another way of installation, e.g. via python venv virtual environment.
  b. Start the spikeNburst GUI with
    spikenburst
    ***Note:*** In all parts of the GUI, hovering with the mouse will show hints for buttons, settings, recordings, etc.
    Troubleshooting: See problem 7 and problem 8.
17. Load spike train data:
  a. Load input data by clicking on the button on the top left with a symbol for opening files (e.g., ‘↥’). A dialog box for file selection appears.
  b. Navigate to the folder, where the input files are stored and select either the single npz or bxr files or folders containing those files.
  c. Confirm the selection by clicking on the button labeled ‘OK’.
  d. Then, the files are loaded into the application and shown in the table (Figure 3).
18. Select recordings to be analyzed by pressing Shift or Ctrl key on the keyboard and selecting items with the mouse pointer.
19. Select active units:
  a. Click on the button labeled ‘Active’.
  b. In the dialog box choose the settings for active units (Table 2, Spikes/Active units; Figure 4A).
  c. Click on the button labeled ‘OK’.
  d. The columns labeled ‘Active’ and ‘Spikes’ will be updated with the number of active units and total number of spikes, respectively.

**CRITICAL:** Make sure to select proper settings. In particular, the choice, whether spike-sorted units shall be used, depends on the type of input files used.

20. Run spike synchrony estimates by clicking on the button labeled ‘Synchrony’.

***Note:*** Depending on the chosen method, the parameters can be adjusted (Table 2, Synchrony; Figure 4B). To use parallelization and speed up the process, increase the number of parallel processes.

**CRITICAL:** If multiple measures of synchrony are to be investigated, repeat the step for each method.

**CRITICAL:** For recordings with many (∼1000) active units the calculation of synchrony measures can take very long or may fail to complete. Choose STTC for those recordings.

21. Detect bursts:
  a. Click on the button labeled ‘Bursts’.
  b. In the ‘Burst detection’ dialog box, select proper settings.
    ***Note:*** Depending on the chosen method, the parameters can be adjusted (Table 2, Bursts; Figure 4C).
  c. After clicking on the button labeled ‘OK’, wait some seconds for the analysis completion.
22. Detect network bursts:
  a. Clicking on the button labeled ‘Network bursts’.
    ***Note:*** This can be only done if step 21 was run before.
  b. Adjust parameters like in step 21 (Table 2, Network bursts; Figure 4D).
23. Plot summary figures:
  a. Clicking on the button labeled ‘Plot’.
  b. Adjust parameters (Table 2, Plotting).

**CRITICAL:** Make sure to select a proper location for the output files and the preferred plot format.

**CRITICAL:** The option ‘Plot settings file’ should usually be kept empty. It can be used to specify advanced settings (see HYPERLINK https://codeberg.org/spiky/spikenburst/src/branch/main/plot_settings.txt).

24. Export the results into tables or python-pickled files by clicking on the button labeled ‘Export’. Adjust parameters (Table 2, General).

**CRITICAL:** Make sure to select a proper location for the output files and the preferred export format.

25. Compare different recordings depending on the conditions ‘Cond. A’ and ‘Cond. B’ that can be modified in the columns of the table in the GUI.

***Note:*** This is an experimental feature that produces some comparison plots. It is recommended to use the exported table files from step 14 to produce plots and calculate proper statistics for publication.

### Use nicespike as a Python module

**Timing: 10–30 min per recording**

The nicespike tool may be accessed via GUI, as described above, or programmatically within a python script or from within a python console. The following steps demonstrate its usage with python commands.

***Note:*** You may use the nicespike and Kilosort 2 within the docker container using the docker container command execution with.

docker exec -it nicespike python

26. Inside of a python file or a python console, import from the nicespike module with.

from nicespike import settings, set_value, SpikeSorting

27. Options may be adjusted with the function set_value, e.g.,

set_value(settings['General']['Number of parallel processes'], 8)

set_value(settings['Spikes']['Minimum firing rate'], 0.05)

set_value(settings['Network bursts']['Minimum number of units'], 5)

set_value(settings['Synchrony']['STTC: time window'], 0.5)

28. A sorting object is created by.

sorting = SpikeSorting(path='brw/div_21_21_chip_A.brw',

output_dir='output/div_21_21_chip_A',

settings=settings,

verbose=True)

29. Read the input file and apply the filtering with.

sorting.read_and_filter()

30. Run the spike sorting using Kilosort with.

sorting.run_kilosort2()

31. Extract the wave forms with.

sorting.extract_waveforms()

32. Optionally, export the npz files, which might be used by spikeNburst tool, with.

sorting.export_npz()

33. Run the spike train analysis (burst and network burst detection, synchrony) with.

sorting.spikenburst_analysis()

34. Plot and export with.

sorting.spikeinterface_plot()

sorting.spikenburst_export()

### Use spikeNburst as a Python module

**Timing: 20 min per recording**

The spikeNburst tool may be accessed via GUI, as described above, or programmatically within a python script or from within a python console. The following steps demonstrate its usage with python commands.

35. Inside a python file or a python console, import the spikeNburst module:

import spikenburst

36. Create a spike analysis object, specifying parameters by.

sa = spikenburst.SpikeAnalysis('npz/div_21_21_chip_A.npz',
     use_spike_sorted_units=True,
     min_firing_rate=0.05)

37. Available parameters for all classes and functions are documented in the help text, e.g., accessible by.

help(spikenburst.SpikeAnalysis)

38. Calculate various synchrony measures with.

sttc = sa.sttc(dt=0.5)

phase_synchrony = sa.phase_synchrony()

spike_distance = sa.spike_distance(n_bin=120)

39. Run the burst detection on the spike analysis object with.

ba = sa.burst_detection('pasquale')

40. Run the network burst detection on the burst analysis object created with.

nba = ba.network_burst_detection('pasquale', min_n_entity=5)

41. Plot the results with given prefixes with.

sa.plot('ouput/div_21_21_chip_A/s_')

ba.plot('ouput/div_21_21_chip_A/b_')

nba.plot('ouput/div_21_21_chip_A/n_')

42. Export the analysis results from the network burst analysis object with.

nba.export('ouput/div_21_21_chip_A.ods')

***Note:*** This will also export the results from the spike and burst analysis.

## Expected outcomes

As illustrative example we recorded a dataset of spontaneous electrophysiological activity of mESCs-derived cortical neurons at two (DIV 21+14) and three (DIV 21+21) weeks after the end of the cortical differentiation. In this section, we demonstrate the effectiveness of the protocol in quantifying synchrony and burst dynamics at both unit and network levels and identifying somatic and dendritic features of single neurons. First, we provide information about the experimental design of the protocol and the tools nicespike and spikeNburst. Second, we elaborate on the need for spike sorting and show example plots that are extracted using the protocol. Third, we introduce a potential follow-up analysis using the exported data from the protocol, in particular we analyzed the bursting behavior and network of units in a recording. We conclude this section with an in-depth explanation of the methods that our protocol uses.

### Experimental design

**Figure 5:**
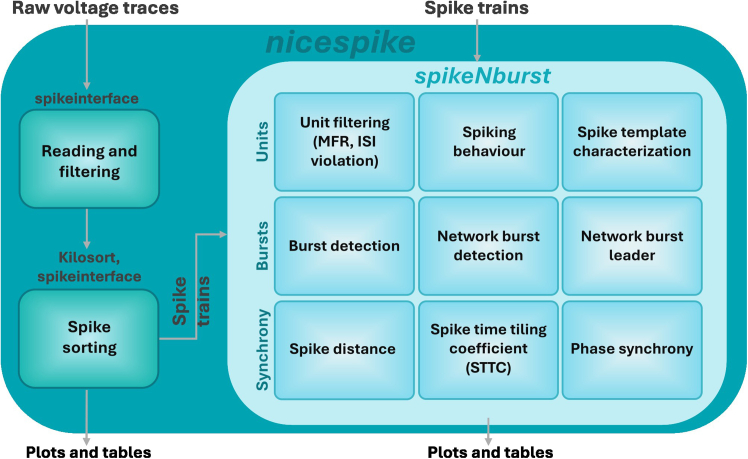
Overview of the experimental design Raw voltage traces are analyzed by nicespike and spike trains by spikeNburst.

In this protocol, we introduce two python-based tools. Depending on the need for spike sorting the nicespike or the spikeNburst tool may be chosen (Figure 5). While spike trains are directly examined by the spikeNburst tool, raw voltage traces are processed by the nicespike tool that ultimately plots and exports the analysis results for each recording, internally using the spikeNburst tool as a python module. Both tools are implemented with a GUI and can be accessed programmatically as python modules. In the above steps, we describe the installation (before you begin) and usage (step-by-step method details) of both tools.

We implemented an analysis pipeline including spike sorting with template matching and the subsequent spike train analysis within the nicespike tool. We used the spikeinterface package^7^ for accessing the brw files (raw voltage recordings by the Brainwave software). For this, we co-authored the brw file reading in the python-neo tool.^17^ Firstly, the raw voltage traces of electrophysiological recordings are filtered. We implemented an automatic channel removal for broken or very noisy channels that removes signals characterized by constant consecutive values for at least 10 samples or by standard deviation (SD) > 1.7 times the median of SD of all channels. Broken channels, determined from previous recordings, are removed, and the raw traces are band-pass-filtered from 300 to 6,000 Hz. Then, spike sorting is done using Kilosort,^18^ running on GPU. The spike-sorted unit spike traces are then exported to custom npz files that are further analyzed internally by the spikeNburst module.

The spikeNburst tool was developed to provide accessible and advanced analysis of spike trains. Our demonstration supports input from both bxr files generated by the Brainwave software and custom npz files (NumPy data archive format, used internally by nicespike). The tool incorporates multiple analytical methods. First, neuronal units can be filtered using the inter-spike-interval (ISI) violation method.^19^ Second, methods for burst and network burst detection are implemented, ranging from basic approaches^20^ to advanced algorithm,^21^ enabling detailed investigation of bursting behavior. Third, a variety of unit synchrony measures are available, including spike distance,^24^ the spike-time tiling coefficient (STTC)^11^ and phase synchrony,^25^^,^^26^^,^^27^ implemented using the burst_sync package.^12^

Both tools include easy exporting as tabular data and basic plotting functionality of the calculated parameters.

### Spike sorting is essential for neurons recorded on HD-MEA devices

**Figure 6:**
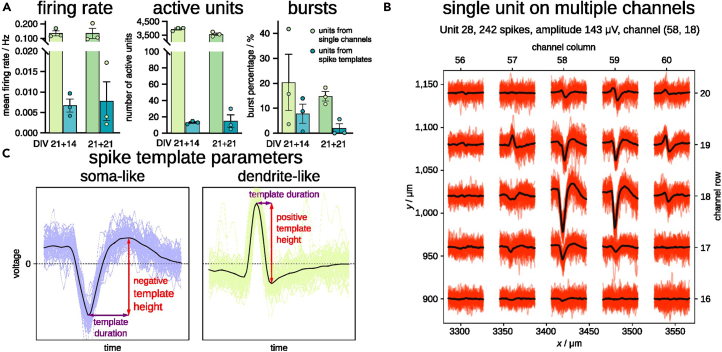
Spikes for single channels and for units from spike-template sorting in E14-mESC-derived neuronal cultures (A) Parameters of recordings in three recordings at DIV 21+14 and three recordings at DIV 21+21 using units from single channels and spike-template sorting. The height of the bars indicates the mean and the error bars represent the standard error of the mean. (B) An example unit that has a matched template with somatic (negative voltage peak) and dendritic (positive voltage peak) parts spanning over at least 200 μm. (C) Estimate of spike template height and duration for soma-like and dendrite-like units.

We analyzed the raw recordings with the described tools using two types of spike detection: 1. the single-channel spike detection from Brainwave, and 2. spike-template matching with Kilosort within the nicespike tool. Subsequently, we performed spike, burst and synchrony measurement using spikeNburst. We observed that the mean firing rate and the number of active units was over-estimated by the single-channel spike detection by one order of magnitude, and more bursts were detected than for units from spike-template matching (Figure 6A). Because the activity of a neuron may spread on multiple channels in HD-MEA devices, we expected a misleading higher number of active units and activity for single-channel spike detection. By contrast, the sorting by spike-template matching, characterized the neuron’s spatial footprint, identifying the highest spiking activity of a single unit captured by a central channel and the traces of spike activity on the surrounding channels (Figure 6B, created within nicespike). Further, the shape of the spike templates can be used to classify the units into soma-like and dendrite-like units (Figure 6C). Particularly, spike templates where the absolute minimum voltage is greater than the maximum voltage are classified as soma-like with an associated negative template height, while dendrite-like units have a positive template height.^28^

**Figure 7:**
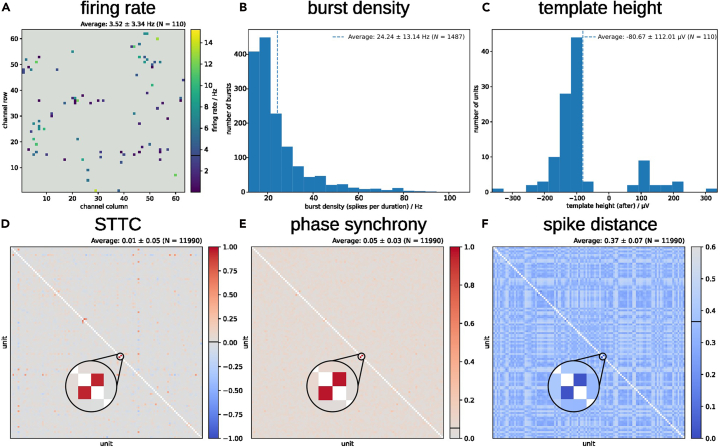
Characterization of a WT-B1-mESC-derived neuronal culture at DIV 21+21 after spike-template sorting using spikeNburst (A) Firing rate of *N* = 110 active units at their locations inside the culture. (B) Burst density of *N* = 1,487 bursts. (C) Unit template height of 19 dendrite-like and 91 soma-like units. (D–F) Various synchrony measures for pairs of units: STTC with Δ*t* = 0.05 s (D), phase synchrony (E) and spike distance with *N*bin = 120 (F). *N* = 2 × number of unit pairs.

We show an example of automated plots produced by spikeNburst, characterizing a WT-B1-mESCs-derived neuronal culture. We found 110 active neurons with few units with firing rates of up to 15 Hz (Figure 7A) and mean burst density, which is defined as the total number of spikes per burst duration, of 24 ± 13 Hz (Figure 7B). 91 units were classified as soma-like, and 19 units were found to be dendrite-like (Figure 7C). To assess the degree to the neuron-neuron interaction, different synchrony measures have been calculated identifying the rhythmicity of groups of neurons acting together (Figures 7D–7F). The STTC and the phase synchrony are 0 for no synchrony and 1 for high synchrony, while the spike distance is 0 for the highest synchrony and greater than 0 for low synchrony. In the culture analyzed, the majority of units were found to be non-synchronized. Two units (with identifiers 26 and 28) showed high synchrony across all three calculated synchrony measures.

### Network analysis

**Figure 8:**
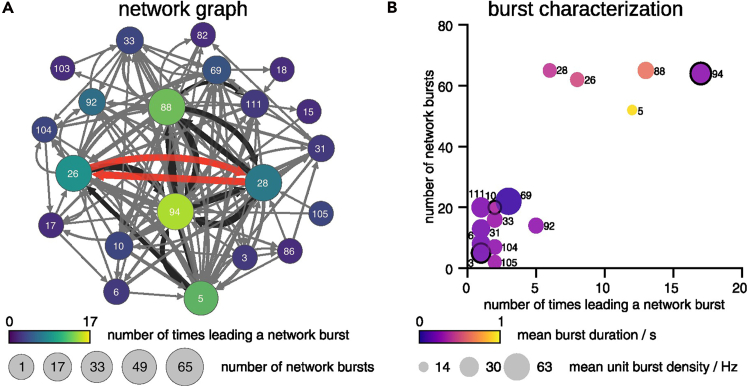
Network bursts in a WT-B1-mESC-derived neuronal culture at DIV 21+21 (A) Network graph determined by *N* = 21 units (nodes) participating in 76 network bursts. The directed edges are defined by one unit preceding another inside a network burst, with their width indicating the degree of connectivity. The edges highlighted in red correspond to the connection of the two units found highly synchronized (Figures 6D–6F). The node’s size indicates the number of times a unit participates in a network burst, the node’s color the number of times a unit leads a network burst (burst leader), and the node’s label the unit’s identifier. (B) Burst characterization for *N* = 15 burst leaders. The point’s size indicates the unit’s mean burst density, the point’s color the unit’s mean burst duration, and the point’s label the unit’s identifier. The three units identified as dendrite-like are marked with black border.

Using the parameters reported in the exported data generated by the protocol, we analyzed firing activity, neural connection and information processing of the units in the network of the examined WT-B1-mESC-derived neuronal culture. We found a group of 21 units that show a network bursting activity. Then, we identified 15 units acting as burst leaders, which means that they initiate network bursts triggering and driving the network population activity. Leveraging the timing correlation between units, we estimated the nodes connectivity, the direction in number of incoming and out-coming edges, and the network hubs such as the units with the identifiers 5, 26, 28, 88 and 94 (Figure 8A). We noticed that the two most synchronized units with the identifiers 26 and 28 (Figures 7D–7F) were found highly connected also in the network analysis (edges marked in red in Figure 8A). We then investigated the bursting behavior of the 15 network burst leaders in dependency on how often they lead a network burst and how often they participate in network bursts (Figure 8B). We observed 5 units with particular high number of network bursts, correlated with high number of times leading a network burst. Interestingly, 4 of these 5 units showed higher mean burst duration with lower burst density and only one unit, the unit with identifier 94, was characterized by more dense, but short-lasting bursts. Interestingly, the calculated spike-template height of this unit suggests classification as a dendrite-like unit while the other 4 units were classified as soma-like.

In summary, spikeNburst and nicespike represent a step forward in the field of *in vitro* and *ex vivo* electrophysiology, enabling researchers to efficiently analyze complex HD-MEA datasets and uncover detailed neuronal dynamics. By addressing critical gaps in current analytical approaches, these tools lay the groundwork for future innovations in the study of neural systems and their applications in neuroscience and bioengineering.

### Methods implemented by nicespike and spikeNburst

The methods implemented are summarized in Table 1 and the parameters of the various analyses that can be adjusted using the GUIs are described in Table 2.

#### Interface to electrophysiological raw voltage recordings

Spike sorting is essential for the analysis of HD-MEA voltage traces. The tool spikeinterface^7^ provides interfaces for different spike sorters and access to various input file formats from multiple manufacturers via the python-neo package.^17^ For the recording system and software we used Brainwave.^13^ There was no open-source tool for reading the raw recordings. Therefore, we implemented the reading of raw voltage traces as well as traces that have been recorded in a sparse format^39^^,^^41^ Further, the protocol uses spikeinterface as a python module to bandpass-filter the voltage traces (300–6,000 Hz) and to exclude noisy or broken channels.

#### Spike sorting

Raw voltage traces are recorded from thousands of electrodes, also called channels, in HD-MEA systems. Because of typical small electrode distance of tens of micrometers, neuronal units can span over multiple channels. Also the sensitivity of these electrodes allows recording multiple units from single channels that may have distinct spiking features. Spike template matching is a standard procedure, well performed by Kilosort,^18^ to circumvent these issues and to firstly detect spikes and secondly to assign them to the individual units. By default, Kilosort 2 is used, since it works well for static recordings and drift is minimum in *in vivo* and *ex vivo* samples. The spike sorting step is run through spikeinterface, which supports several other spike sorting methods and thus would make it easy to swap in a different algorithm if needed.

#### Unit filtering

Additionally to filtering units by setting a minimum level of firing rate (MFR), quality assessment of units was implemented using the inter-spike-interval (ISI) violation method.^19^ This method allows decreasing the likelihood of false negatives for inadequately sorted units.

#### Unit characterization

Different parts of the same neuron have different spike features.^28^ We implemented the characterization of units depending on the unit spike template, which is the mean of all voltage traces at a spike within a time window of 1 ms before and 2 ms after the spike. From the shape of the spike template we estimated the mean amplitude and duration of spikes for a unit. By amplitude analysis, we classified soma-like or dendrite-like units’ signals depending on the spike template shape of the central channel that has the highest voltage difference. Spike templates where the absolute minimum voltage is greater than the maximum voltage are classified as soma-like with an associated negative template height, while dendrite-like units have a positive template height greater than the negative one (Figure 6). The mean spike duration, also known as peak latency, was applied to identified regular-spiking and intrinsically bursting neurons. The unit spike duration parameter, coupled with optical imaging analysis, was previously applied to identify in a culture putative glutamatergic pyramidal neurons, which show broader spikes, and putative GABAergic interneurons, which show fast spikes.^29^^,^^30^^,^^31^

#### Burst, network burst, and burst leader detection

Different firing properties and excitability of neurons significantly contribute to neural connection and information processing in the network.^42^ Bursting and network bursting are complex and organized cellular behaviors that, in *in vitro* neuronal cultures, can occur spontaneously and characterize the network with brief periods of high firing rates.^43^ Within a neural network, high-specialized bursting neurons act as leaders, firing at the beginning of each burst and triggering and driving the network population activity.^22^^,^^23^

We re-implemented previously described methods that relied on Matlab code (Spycode^14^) in python to enhance the ability to characterize burst and network burst activity. Units are bursting when they have many spikes in short time periods. A simple burst detection^20^ using only two parameters, the maximum ISI for spikes and minimum number of spikes (Table 2, Bursts), and a more advanced method with an approach for self-adapting parameters^21^ were implemented. Further, we implemented a hybrid method that uses the calculation of the ISI thresholds by the advanced method, but the burst detection with these thresholds as maximum ISI according to the simple method.

Networks bursts are subsequently defined as parallel bursting of multiple units and their detection is defined analogously. To adapt to the high number of electrodes in HD-MEA devices, we replaced the parameter of the minimum percentage of recording electrodes involved in a network burst by an absolute minimum number of spiking units. Additionally, we implemented an algorithm to merge network bursts that are overlapping or have small intervals between end of one and start of another network burst.

#### Synchrony

Alternatively to studying the bursting behavior of single units, various synchrony measures have been proposed that measure relations of spike trains of two units. We used the implementation of the burst_sync package^12^ (with minor fixes for latest python versions) to calculate the synchrony of two units. First, the spike distance^24^ is a measure that is close to zero for synchronous firing. In the used implementation it includes only one parameter, which is the number of bins for the dissimilarity quantification between spike trains (Table 2, Synchrony). Second, the spike-time tiling coefficient (STTC)^12^ was introduced with certain necessary and desirable properties in mind, such as symmetry, robustness for firing rate variations, recording duration and small parameter variations, etc. There is one parameter, the time window Δ*t* that can be adjusted within the proposed tools (Table 2, Synchrony). Third, the phase synchrony and the related global synchrony^25^^,^^26^^,^^27^ is a measure of synchrony that does not imply the use of any parameter. It has been demonstrated that different synchrony measures characterize different aspects of synchrony of spike trains.^12^

## Limitations

The protocol provides open-source tools and step-by-step instructions for the analysis of raw electrophysiological voltage recordings from HD-MEA systems and spike trains. The integrated GUIs and the possibility to be used as python modules ensure accessibility for diverse research needs from high-throughput batch analyses to individual experiment visualization.

However, some limitations about the equipment or basic informatics knowledge may arise to properly install the tools of the protocol. In detail, the type of computer used to run the analysis has to provide sufficient working memory and CPU power. A GPU supported by CUDA is necessary to run the spike sorting with nicespike. Moreover, some basic knowledge of the Linux terminal is required to run the commands and work on the directory during the installation of the tools. To overcome this initial limit, the protocol provides step-by-step support in the basic computer commands. Some programming skills are required if the tools shall be used for inputs from different recording systems. To note is that the use of the GUI of spikeNburst and nicespike does not require programming skills. Bypassing these limitations, the clear workflow and the data obtained by the tools allow researchers to respond to varying input datasets by choosing among the parameters in a transparent and reproducible manner and encompass comprehensive analysis at every stage of the pipeline.

## Troubleshooting

### Problem 1

When opening nicespike a connection error may happen when the docker container is not running or the specified port is wrong (step 1).

### Potential solution

Check that the docker container is running or restart it if necessary, e.g., by executing.

docker restart nicespike

Verify that the specified port in the installation matches the port in the URL for opening nicespike (see lines 9–10 in docker-compose.yml).

### Problem 2

The input files may not be found, if the folder is not mounted correctly to the docker container (step 9).

### Potential solution

Check that the input folder is correctly mounted within the docker container (see lines 11–13 in docker-compose.yml).

### Problem 3

Sometimes, in particular for remote connections, the archive is created, but the user is not prompted to save it (steps 12, 13 and 14).

### Potential solution

Check manually (not from within nicespike) the contents of the output directory and search for a file with name ‘plots.zip’, ‘export.zip’, or ‘logs.zip’.

### Problem 4

Selected recordings may not be in the archive, when recordings were not analyzed or there were errors, or no units found (steps 12 and 13).

### Potential solution

Check if the third column (‘Analysed’) in the table of the nicespike tool for the recording has a tick. If not, run the analysis on that recording. If there is an error message appearing, check the logs for this recording (either within the error message or via the log file export on this recording).

### Problem 5

Some expected plots may be missing, if they were disabled in the settings (step 12).

### Potential solution

Check the plotting settings.

### Problem 6

The GUI is in an unresponsive or frozen state, because deletion of many big files takes time (step 15).

### Potential solution

Wait some time. Restart the docker container.

### Problem 7

The spikeNburst executable is not found if the installation was not successful or the environment as not activated (step 16).

### Potential solution

Activate the conda (or python-venv) environment and make sure that the installation inside the environment completed successfully.

### Problem 8

The spikeNburst GUI may not show up if some dependencies are missing, e.g., GTK (step 16).

### Potential solution

Install the missing dependencies. It is recommended to use a conda environment for their installation.

## Resource availability

### Lead contact

Further information and requests for resources and reagents should be directed to and will be fulfilled by the lead contact, Dr. Valter Tucci (valter.tucci@iit.it).

### Technical contact

Technical questions on executing this protocol should be directed to and will be answered by the technical contact, Dr. Robert Wolff (mahlzahn@posteo.de).

### Materials availability

This study did not generate new unique reagents.

### Data and code availability

Original data have been deposited at G-Node: https://gin.g-node.org/spiky/Wolff_et_al_2025_spiky_data (https://doi.org/10.12751/g-node.9sypk7). They include brw files with the raw voltage traces, bxr files with the spike and burst trains from single channels, npz files with the spike trains for spike-sorted units, ods files with the analysis exports, network edges data files, and source data of the figures.

The source code for nicespike and spikeNburst is available at Codeberg at https://codeberg.org/spiky/nicespike (https://doi.org/10.12751/g-node.2jqfxi) and https://codeberg.org/spiky/spikenburst (https://doi.org/10.12751/g-node.5n7v18), respectively.

## Acknowledgments

This study was funded by the European Union – NextGenerationEU and by the Italian Ministry of University and Research (MUR), National Recovery and Resilience Plan (NRRP), Mission 4, Component 2, Investment 1.5, project “RAISE – Robotics and AI for Socio-economic Empowerment” (ECS00000035). V.T. and M.C. are part of RAISE Innovation Ecosystem.

We thank Prof. K. John McLaughlin, Columbus, Ohio, US, and Dr. T. Bouschet, CNRS, Montpellier, France, for kindly authorizing the use and providing us with the WT-B1 mESC line used in this protocol.

## Author contributions

R.W., A.P., and V.T. developed the protocol. A.P. performed the experiments. R.W. implemented the software and algorithms. A.P.B. contributed to the software for the processing of input files from raw electrophysiological recordings. M.C. and V.T. supervised the study. R.W., A.P., and V.T wrote the manuscript with contributions from A.P.B. and M.C. R.W. and A.P. contributed equally.

## Declaration of interests

The authors declare no competing interests.
